# The INDICATE Knee expectations survey detects general patient treatment goals for total knee arthroplasty and the influence of demographic factors on patients expectations

**DOI:** 10.1007/s00167-022-07012-4

**Published:** 2022-06-10

**Authors:** Felix Wunderlich, Lukas Eckhard, Matthias Büttner, Toni Lange, Jürgen Konradi, Ulrich Betz, Philipp Drees, Jörg Lützner

**Affiliations:** 1 Department of Orthopedics and Traumatology, University Medical Center of the Johannes Gutenberg University Mainz, Mainz, Germany; 2 Institute of Medical Biostatistics, Epidemiology and Informatics, University Medical Center of the Johannes Gutenberg University Mainz, Langenbeckstrasse 1, 55131 Mainz, Germany; 3 Center for Evidence-Based Healthcare, University Hospital and Medical Faculty Carl Gustav Carus, TU Dresden, Dresden, Germany; 4 Institute of Physical Therapy, Prevention and Rehabilitation, University Medical Center of the Johannes Gutenberg University Mainz, Mainz, Germany; 5 University Center of Orthopedics, Trauma and Plastic Surgery, University Hospital Carl Gustav Carus Dresden, TU Dresden, Dresden, Germany

**Keywords:** Total knee arthroplasty, Patient expectation, Impairment, Treatment goal, Satisfaction, INDICATE Knee Score

## Abstract

**Purpose:**

Post-operative outcome after total knee arthroplasty (TKA) in the treatment of end-stage osteoarthritis correlates strongly with pre-operative impairment-driven patient treatment goals. However, a clinical tool for measuring patient treatment goals in correlation to impairments is still missing, which impedes patient-oriented indication in TKA.

**Methods:**

Patients scheduled for TKA were recruited in four German hospitals. All patients were handed the INDICATE Knee Score pre-operatively. The score contains 31 treatment goals with respective impairments, subdivided into seven categories. They were asked to rank all treatment goals and impairments on a 3-point scale. Treatment goals and impairments were then checked for frequency of occurrence. Correlation of goal and impairment was tested. Analysis for associations of treatment goals and different cohort characteristics (age, sex, BMI) was conducted.

**Results:**

1.298 patients were included in the study. Seven treatment goals were categorised as “main goal” from more than 90% of all patients (“knee pain”, “range of motion”, “walking distance”, “overall physical function”, “climbing stairs”, “quality of life”, “implant survival”). Comparing age groups, there were significant associations towards higher expectations regarding working, physical and sports related treatment goals in younger patients (< 65y) (“ability to work” (*P* ≤ .001), “sports activities” (*P* ≤ .001), “sex life” (*P* ≤ .001), “dependence on help of others” (*P* = .015), “preventing secondary impairment” (*P* = .03), “dependence on walking aids” (*P* = .005)). Higher BMI resulted in increasing relevance of “weight reduction” (*P* ≤ .001), “climbing stairs” (*P* = .039) “global health status” (*P* = .015) and “long standing” (*P* = .007) as a “main goal”. Analysis for differences in treatment goals regarding sex showed women choosing more treatment goals as “main goals” than men.

**Conclusion:**

Seven treatment goals which were expected by > 90% in our collective can be classified as general treatment goals for TKA. Demographic factors (age, sex, BMI) were significantly associated with patients’ expectations for TKA. We conclude physicians should clearly assess their patients’ demands prior to TKA to maximise post-operative outcome.

**Level of evidence:**

Prognostic Level III.

## Introduction

Total knee arthroplasty (TKA) is a common and frequent orthopaedic procedure for the treatment of end-stage knee osteoarthritis (OA), and its cost-effectiveness and improvement of quality of life are well proven [14]. A large number of studies have shown that patients after TKA are satisfied in most cases, but a relevant amount remains unsatisfied [4, 29]. Among other factors (i.e. surgical or patient related factors), fulfillment of patients’ expectations has been linked with post-operative satisfaction [20, 28]. It is known, that patients tend to have overly optimistic pre-operative expectations in TKA [23], and that patients dissatisfied after TKA were most likely those who had unfulfilled expectations [4, 8].

Consequently, it seems highly necessary to discover patient expectations prior to surgery for adequate shared decision-making (SDM) in indication for TKA, and hereby maximising post-operative satisfaction. Many instruments to measure expectations in orthopaedic procedures are physician derived [30]. To gain more objective, patient-derived data on patients’ expectations, the most widespread instruments for measuring pre-operative expectations are the patient-derived Hospital for Special Surgery (HSS) Hip and Knee Replacement Expectations Surveys, published by Mancuso et al. in 1997 and 2001, respectively [5, 22]. In these questionnaires, Mancuso et al. did not observe the association between patients’ actual impairments and their expectations. However, studies have shown that severe symptoms are a significant predictor for patient expectations in total joint arthroplasty [31]. Despite the high relevance of patients’ impairments for their expectations, a clinical instrument for measuring expectations in correlation to impairments is still missing [30].

Furthermore, expectations may vary between geographical and cultural regions. Therefore, a questionnaire based on previously published known expectations and patient-defined treatment goals in relation to their respective impairments has been developed [12].

The objective of this study was to assess patients’ pre-operative impairments, treatment goals, and their correlation in a cross-sectoral large cohort using this new questionnaire. It was hypothesised that demographic factors (age, sex, BMI) significantly influence pre-operative treatment goals in TKA.

## Materials and methods

Prior to this study, the INDICATE Knee Score questionnaire containing a set of consensus-based treatment goals was developed via a 3-stage Delphi study [17, 18]. Based on the previously published known expectations for TKA, the patients were able to add further treatment goals during the Delphi process. The Delphi survey technique is a common method to achieve a group consensus across disciplines in which individual opinions are combined into group consensus [13].

**Fig. 1:**
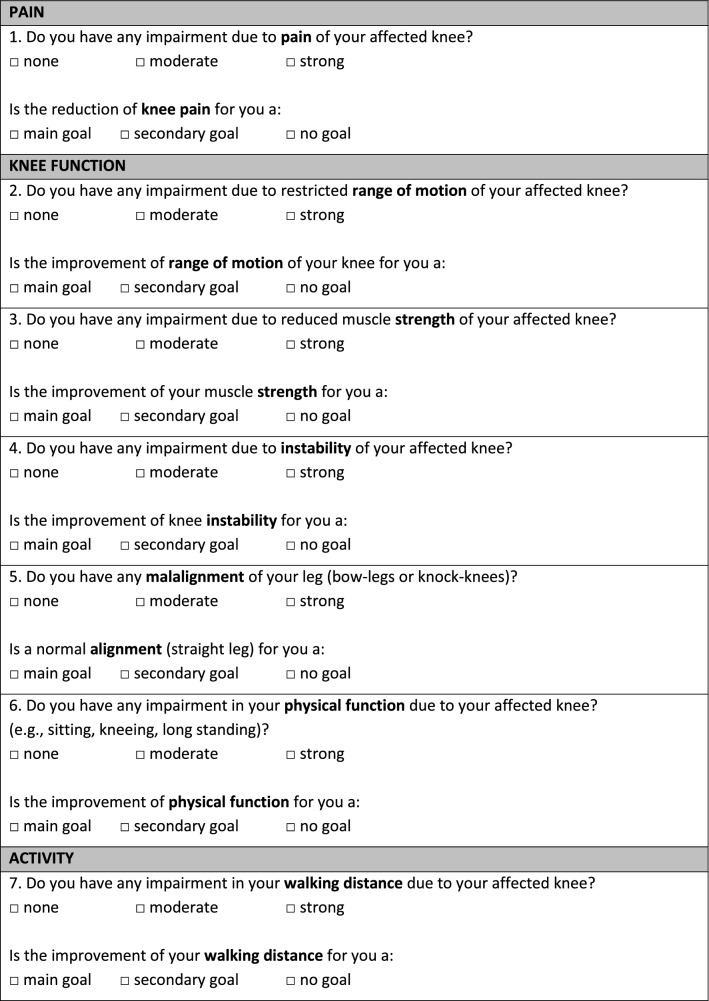

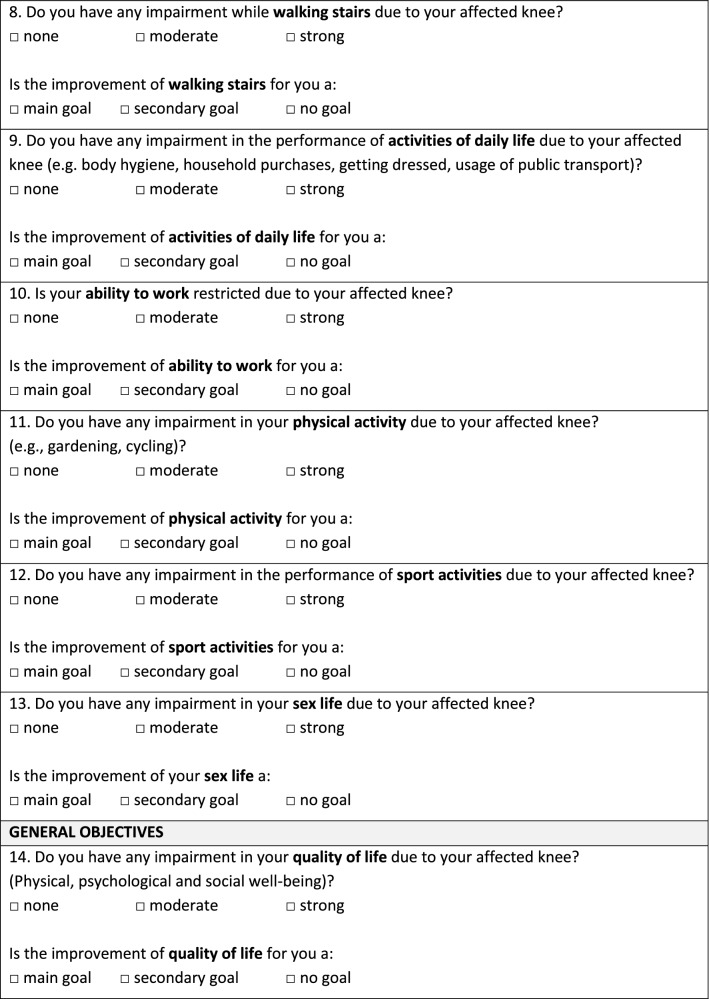

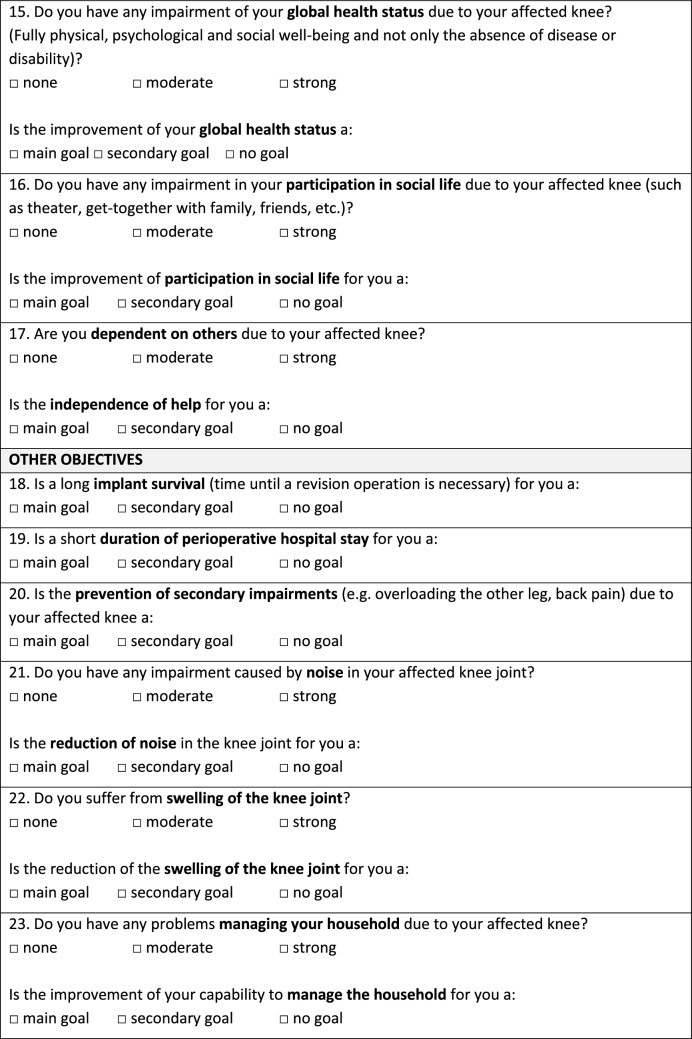

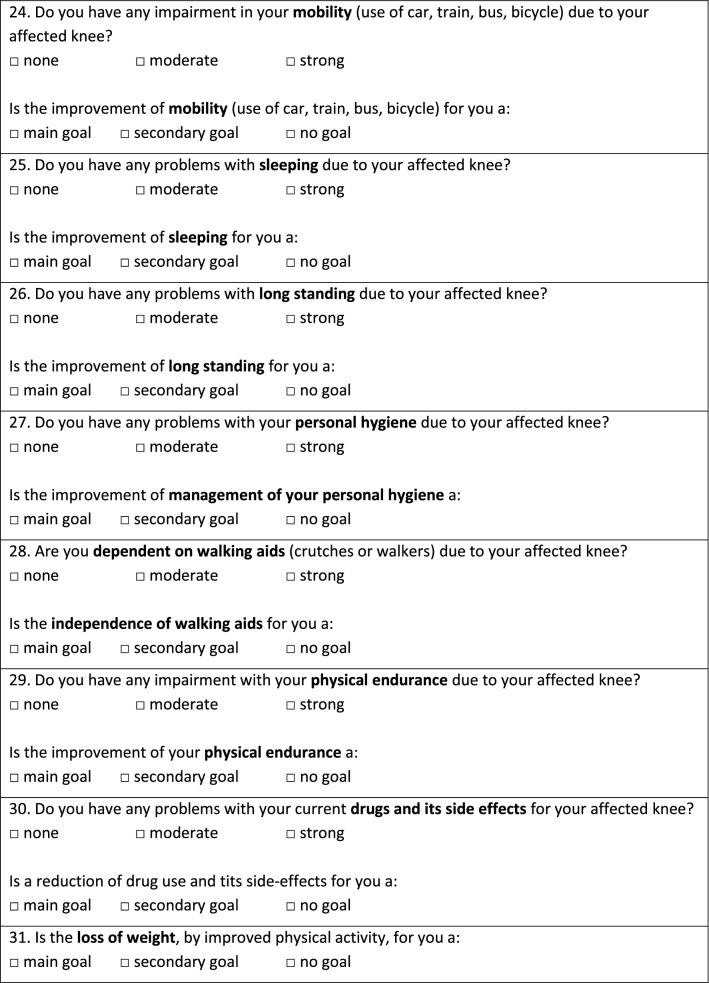
Questionnaire

As shown in Fig. 1, the questionnaire contains 31 patient treatment goals assorted into seven categories (symptoms, physical function, activities of daily life, quality of life, physical activity, coping strategies, and various issues). Patients were asked to rank all proposed treatment goals on a 3-point Likert scale (“main goal”, “secondary goal”, “no goal”). “Main goals” were defined as an outcome that must be reached through TKA, otherwise the procedure would not be considered successful by the patient. “Secondary goals” were desirable goals but were not necessary for the success of the surgery. “No goals” were defined as unimportant. Patients could select as many goals as “main goals”, “secondary goals” or “no goal” as they desired. Additionally, the current subjective perception of impairment was asked for each treatment goal via a 3-point scale (“no impairment”, “moderate impairment”, “severe impairment”).

After approval from all ethics committees in the participating states, all patients older than 18 years that met standardised criteria for surgery [27] and were scheduled for TKA due to advanced OA of the knee were included. Exclusion criteria were German language inability, noncompliance of signing a written consent, life expectancy less than 1 year as judged by the treating physician, and any health factors that would preclude elective surgery.

Participants eligible for the study were recruited in four different types of German hospitals: two tertiary referral university hospitals, one orthopaedic specialised arthroplasty hospital and one regional hospital. A large sample of patients were included in this study via the PROMISE Trial [3]. All eligible patients were asked to participate and the questionnaire was handed out after informed consent,

Data collection was conducted in an outpatient clinic at time of surgery indication 1–8 weeks prior to TKA. Included patients were handed the survey during their outpatient clinic visit. Qualified study nurses on site explained the questionnaire and the participants were asked to complete the survey on their own. The questionnaire was then checked for completion. In addition to the expected treatment goals, baseline data were collected to describe the study population including age, sex, side of surgery, diagnosis, BMI, and socioeconomic data (education, living with partner, working situation). Data were assembled from all study sites and stored pseudonymised in an electronic database.

### Statistical analysis

Patient characteristics, impairments and treatment goals were expressed as mean or percentage values as appropriate. Univariate comparisons of treatment goals and age, sex, and BMI were performed using Chi-Square and Fisher-Exact Tests. Spearman rank correlation was used for correlation of treatment goals and the respective impairments. Ranges for correlation strength were considered as “very strong” (0.80–1), “strong” (0.60–0.79), “medium” (0.40–0.59), “weak” (0.20–0.39) and “very weak” (0.00–0.19) [15]. Post hoc power calculation showed that there was a power above 80% to detect weak correlations given the used sample and an alpha-level of 0.05. All statistical analyses were performed using R (R version 3.5.1, Core Team (2017)) [34].

## Results

1.298 patients with complete data sets were included in the study. Mean age was 67.4 years (SD 9.74), 55% were women. 92.1% of all patients received TKA because of primary osteoarthritis. Side of surgery was nearly evenly distributed (46.3% left vs. 49.2% right) with 59 patients (4.5%) receiving simultaneous bilateral TKA. All baseline data are shown in Table 1.

**Table 1:** Patient characteristics

Variable	
Age (y) (mean [SD])	67.35 (9.74)
Sex	
Male	45.0% (584)
Female	55.0% (714)
Side of surgery	
Left	46.3% (601)
Right	49.2% (638)
Both	4.5% (59)
Reason for surgery	
Primary osteoarthritis	92.1% (1196)
Posttraumatic osteoarthritis	5.0% (65)
Other	2.6% (34)
Missing	0.2% (3)
BMI (kg/m2) (mean[SD])	30.6 (7.4)
Education	
< Ten years	41.1% (533)
Ten years	34.1% (442)
> Ten years	22.3% (289)
Other	0.4% (5)
Missing	2.2% (29)
Living with partner	
Yes	75.0% (974)
No	23.6% (306)
Missing	1.5% (18)
Working situation	
Unemployed / Not working	3.9% (51)
Working	30.0% (389)
Retired	63.3% (821)
Other	0.9% (12)
Missing	1.9% (25)

*BMI* body mass index

### Impairments

**Fig. 2:**
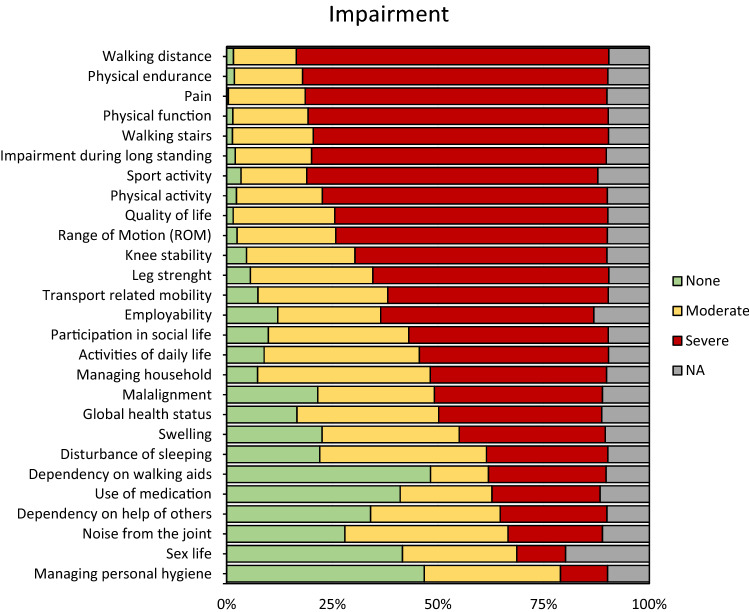
Impairments of patients

The most frequently chosen “severe impairments” were “walking distance”, “physical endurance”, “knee pain”, “overall physical function”, “climbing stairs”, “long standing”, “sports”, and “physical activity”. The impairments “noise from the joint”, “sex life” and “managing personal hygiene” were least frequently chosen as “severe impairment”. One percent of patients (*N* = 13) chose all impairments as “severe impairment”. Distribution of impairments is shown in Fig. 2.

### Expectations

**Fig. 3:**
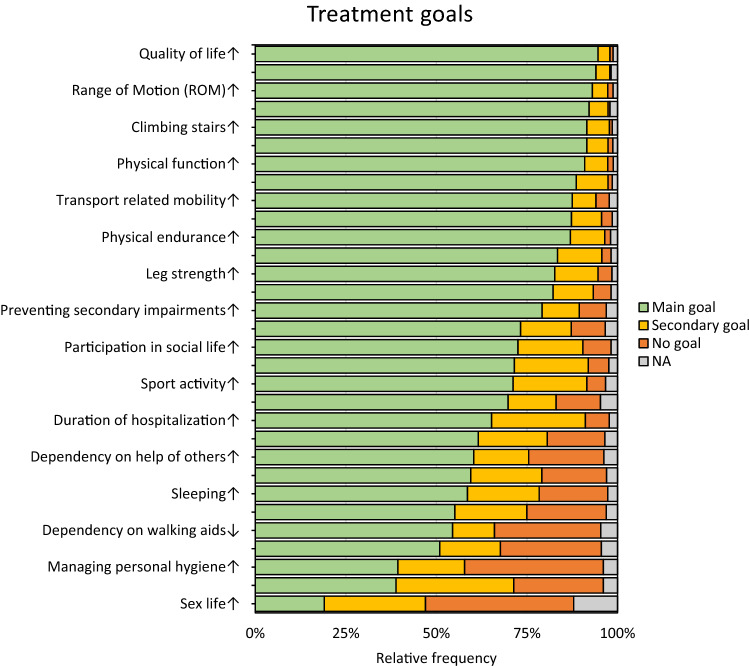
Treatment goals of patients

Evaluation of treatment goals showed seven treatment goals being categorised as “main goal” from more than 90% of all patients (“knee pain”, “range of motion”, “walking distance”, “overall physical function”, “climbing stairs”, “quality of life”, “implant survival”). Thirty-five (2.7%) patients chose all treatment goals as “main goal”. Figure 3 illustrates the ranked proportions of main goal, secondary goal, and no goal in the study population.

Statistically significant differences could be shown between age (< 55, 55–65, > 65 years), sex (male, female), and BMI (< 25, 25–30, > 30 kg/m^2^) and treatment goals (Table 2). When stratified by age, “ability to work” and “sex life” were more often chosen as “main goal” by younger patients (< 55 years). “Weight reduction” showed higher percentages as “main goal” in young patients < 55 years than in patients > 65 years (68.4% vs 48.3%), whereas “sports activities” was more often chosen as “main goal” by patients between 55 and 65 years (70.7% vs 75.9% vs 68.8%). There were also significant differences when stratifying treatment goals by sex, with women choosing more treatment goals as “main goals” than men. The categories “walking distance”, “ability to work”, “sex life”, “physical activities” and “long standing” were more often chosen as “main goal” by men, although without any statistical significance. With increasing BMI (< 25, 25–30, > 30 kg/m^2^), “weight reduction” was more often chosen as “main goal”.

**Table 2:** Percentage of “main goals” chosen in each group and overall statistical significant differences (as p-values*) between “treatment goals” in different subgroups (statistical significant results in bold type)

Category	Age				BMI				Sex		
	< 55 y	55–65y	> 65y	*p*-value	< 25	25–35	> 35	*p*-value	Male	Female	*p*-value
Reduction of knee pain	94.7% (126)	94.5% (380)	93.7% (714)	.454	**89.3% (184)**	**95.4% (456)**	**94.8% (579)**	**.007**	**94.0% (549)**	**94.1% (672)**	**.041**
Range of motion (ROM)	93.2% (124)	91.5% (368)	93.8% (715)	.700	**89.3% (184)**	**92.7% (443)**	**94.6% (578)**	**.047**	**91.3% (533)**	**94.5% (675)**	**.002**
Leg strength	82.0% (109)	82.8% (333)	82.8% (631)	.370	80.1% (165)	82.6% (395)	83.6% (511)	.696	**79.6% (465)**	**85.1% (608)**	**.002**
Knee stability	85.7% (114)	86.6% (348)	88.1% (671)	.417	88.3% (182)	86.6% (414)	87.4% (534)	.758	**84.4% (493)**	**89.6% (640)**	**.002**
Malalignment	**54.9% (73)**	**62.4% (251)**	**62.3% (475)**	**.03**	62.6% (129)	64.0% (306)	59.2% (362)	.619	**57.7% (337)**	**64.7% (462)**	**.001**
Physical function	93.2% (124)	92.3% (371)	89.8% (684)	.290	89.3% (184)	90.2% (431)	92.0% (562)	.140	89.7% (524)	91.9% (656)	.06
Walking distance	87.2% (116)	92.0% (370)	92.0% (701)	.120	88.8% (183)	91.8% (439)	92.1% (563)	.068	92.0% (537)	91.2% (651)	.108
Climbing stairs	91.7% (122)	89.6% (360)	92.5% (705)	.306	**88.3% (182)**	**91.6% (438)**	**92.6% (566)**	**.039**	**90.8% (530)**	**92.2% (658)**	**.044**
Activities of daily life	85.0% (113)	79.9% (321)	82.9% (632)	.766	**77.7% (160)**	**80.1% (383)**	**85.4% (522)**	**.035**	**77.6% (453)**	**86.0% (614)**	** < .001**
Ability to work	**85.0% (113)**	**76.1% (306)**	**63.8% (486)**	** < .001**	64.6% (133)	68.4% (327)	72.5% (443)	.164	70.7% (413)	69.0% (493)	.067
Physical activities	88.0% (117)	89.1% (358)	88.6% (675)	.839	84.5% (174)	89.7% (429)	89.0% (544)	.227	88.7% (518)	88.5% (632)	.784
Sport activities	**70.7% (94)**	**75.9% (305)**	**68.8% (524)**	** < .001**	71.4% (147)	72.8% (348)	69.7% (426)	.275	70.7% (413)	71.6% (511)	.454
Sex life	**30.1% (40)**	**25.6% (103)**	**13.6% (104)**	** < .001**	18.0% (37)	17.6% (84)	20.3% (124)	.342	21.4% (125)	17.1% (122)	.054
Quality of life	93.2% (124)	95.3% (383)	94.4% (720)	.274	91.7% (189)	95.2% (455)	95.1% (581)	.328	94.3% (551)	94.8% (677)	.113
Global health status	76.7% (102)	74.4% (299)	72.2% (550)	.307	**67.5% (139)**	**70.5% (337)**	**77.3% (472)**	**.015**	72.1% (421)	74.2% (530)	.319
Participating in social life	72.9% (97)	72.6% (292)	72.4% (552)	.672	**67.5% (139)**	**70.3% (336)**	**75.8% (463)**	**.038**	**67.6% (395)**	**76.5% (546)**	** < .001**
Less dependence on help of others	**56.4% (75)**	**56.2% (226)**	**63.3% (482)**	**.015**	**54.4% (112)**	**56.5% (270)**	**65.1% (398)**	**.011**	**54.8% (320)**	**64.8% (463)**	** < .001**
Implant survival	**88.7% (118)**	**94.8% (381)**	**91.3% (696)**	**.003**	88.8% (183)	92.5% (442)	93.0% (568)	.161	92.0% (537)	92.3% (659)	.57
Short hospitalisation	59.4% (79)	65.7% (264)	66.0% (503)	.450	62.1% (128)	65.9% (315)	65.8% (402)	.478	64.4% (376)	66.0% (471)	.6
Preventing secondary impairments	**82.0% (109)**	**83.6% (336)**	**76.5% (583)**	**.03**	77.7% (160)	77.4% (370)	81.0% (495)	.620	77.1% (450)	81.0% (578)	.206
Noise from the joint	**35.3% (47)**	**42.0% (169)**	**37.8% (288)**	**.035**	35.4% (73)	41.6% (199)	37.8% (231)	.105	38.0% (222)	39.5% (282)	.403
Reduction of Swelling	**63.2% (84)**	**63.2% (254)**	**56.8% (433)**	**.003**	**54.4% (112)**	**57.9% (277)**	**62.4% (381)**	**.044**	**54.1% (316)**	**63.9% (456)**	** < .001**
Managing household	70.7% (94)	68.2% (274)	73.5% (560)	.072	**64.6% (133)**	**70.1% (335)**	**75.0% (458)**	**.016**	**62.7% (366)**	**78.7% (562)**	** < .001**
Transport related mobility	91.7% (122)	86.6% (348)	87.4% (666)	.533	**86.9% (179)**	**84.7% (405)**	**89.9% (549)**	**.036**	**84.4% (493)**	**90.1% (643)**	** < .001**
Sleeping	62.4% (83)	60.7% (244)	56.7% (432)	.246	51.0% (105)	58.25 (278)	61.2% (374)	.100	**52.9% (309)**	**63.2% (451)**	** < .001**
Long standing	85.0% (113)	82.3% (331)	83.7% (638)	.918	**77.2% (159)**	**84.5% (404)**	**84.6% (517)**	**.007**	83.7% (489)	83.2% (594)	.556
Managing personal hygiene	**33.1% (44)**	**35.3% (142)**	**42.7% (325)**	**.028**	33.0% (68)	40.4% (193)	40.6% (248)	.318	38.0% (222)	40.5% (289)	.315
Less dependence on walking aids	**43.6% (58)**	**52.7% (212)**	**57.3% (437)**	**.005**	54.9% (113)	51.7% (247)	56.5% (345)	.389	53.1% (310)	55.6% (397)	.488
Physical endurance	85.7% (114)	84.8% (341)	88.3% (673)	.093	82.5% (170)	88.1% (421)	87.6% (535)	.104	85.5% (505)	87.4% (624)	.242
Less use of drugs	59.4% (79)	51.5% (207)	49.2% (375)	.347	47.6% (98)	50.0% (239)	52.5% (321)	.707	**47.3% (276)**	**53.9% (385)**	**.007**
Weight reduction	**68.4% (91)**	**63.7% (256)**	**48.3% (368)**	** < .001**	**17.5% (36)**	**47.5% (227)**	**73.8% (451)**	** < .001**	**50.7% (296)**	**58.7% (419)**	**.005**

*All *p*-values generated via Chi-Square and Fisher-Exact Test

### Correlations

Positive correlation between impairment and the respective treatment goal could be shown for all categories (Table 3). Only one “very strong” correlation occurred in the category “sex life” (0.80), whereas no category showed a “very weak” (0.00–0.19) correlation.

**Table 3:** Spearman's correlation rank test: treatment goals and impairments

Category	Spearman’s Rho*
Sex life	0.801
Swelling	0.749
Personal hygiene	0.749
Sleeping	0.744
Noises	0.731
Drugs and its side effects	0.691
Leg alignment	0.650
Dependence on walking aids	0.644
Dependence on others	0.638
Ability to work	0.622
Global health status	0.599
Participation on social life	0.593
Managing household	0.523
Strength	0.519
Stability	0.503
Activities of daily life	0.500
Physical function	0.461
Transport-related mobility	0.456
Sport activities	0.444
Long standing	0.436
Walking distance	0.382
Joint mobility	0.356
Climbing stairs	0.353
Physical activity	0.346
Physical endurance	0.335
Quality of life	0.306
Knee pain	0.246

*00–.19 “very weak”; .20–.39 “weak”; .40–.59 “moderate”

60–.79 “strong”; .80–1.0 “very strong”; Sorted order by strength of correlation

## Discussion

The most important findings of this study were the seven treatment goals chosen as “main goal” from over 90% of all patients. Those can, therefore, be considered as generalised treatment goals for TKA. Patients’ expectations for TKA showed significant differences related to demographic factors (age, sex, BMI). Correlation between impairments and the respective treatment goals differed considerably. Overall, expectations were generally high.

This study describes the first implementation of a new, systematically developed, impairment-driven questionnaire (INDICATE Knee Score) [18], containing 31 items to cover a wide range of expectations in patients undergoing TKA. In contrast to the pre-existing patient expectations questionnaires of Mancuso et al. [5], a 3-point rather than a 5-point Likert scale was used, which has a good translation into clinical context and has been shown to be far easier to use, especially by patients with intellectual disability or non-readers [6, 9, 17]. The possibility to put patients’ expectations into context with their existing impairments could achieve better understanding of patients’ frames-of-reference concerning their pre-operative degree of activity. Different frames-of-reference have a significant influence on validity of current expectation surveys, as stated by Hepinstall et al. in 2011 [12]. A strength of this study is the large, heterogeneous sample of patients, based on the recruitment of participants in four different hospitals representing all levels of care. The cohort matches the reported distributions for age, sex, and BMI in TKA in Germany and other countries [1, 2, 10].

Assessing the distribution of patient expectations in our study, Hawker et al. showed similar results in a prospective study in a Canadian cohort [11]. Nevertheless, one has to take into account the previously described influence of ethnicity on expectations in patients undergoing TKA [16, 19]. Well-known treatment goals (e.g. “pain reduction”, “improvement of range of motion”, or “physical ability”) could be confirmed in our cohort. A potentially less known goal with high importance in our study was “long implant survival”. Because it can be presumed that surgeons generally expect a long implant survival, it might not always be addressed in the pre-operative discussion, even though it tends to be from great importance for patients undergoing TKA. This could lead to unfulfilled patient expectations in the post-operative care, if the estimated implant survival cannot be achieved. Unfulfilled expectations have been demonstrated to be a main reason for dissatisfaction after TKA [4], which is even more important under the aspect of overly optimistic pre-operative patient expectations in TKA, as shown by Mannion et al. [23]. The seven most chosen treatment goals discovered in this study should be considered as universal treatment goals and should hence be queried in addition to the patients’ individual treatment goals before surgery. Physicians should mandatorily assess and discuss the probability of their patients achieving these universal treatment goals during SDM and additionally should inquire for further, more individual patient treatment goals to detect special needs. Because of its precise 31 Items, the INDICATE Knee Score seems to be superior for these matters than the currently available expectations questionnaires. Surgeons could then exert a modifying influence on their patients’ expectations during the pre-surgical phase to guide them to more realistic treatment goals and hereby ensure post-operative realisation of expectations to improve overall satisfaction. The questionnaire further could help to close the previously demonstrated gap between patients’ overly optimistic, and surgeons’ realistic expectations [23, 24], which might support dissatisfaction after TKA.

Significant differences were demonstrated in expectations expressed as treatment goals regarding different demographic factors (age, sex, BMI). Previous literature is contradictory regarding the influence of age and sex on expectations [19, 21], with newer studies describing female and older patients having lower expectations on the outcome of TKA [12, 31]. This could not be confirmed in our study, with women having generally higher expectations on TKA and choosing more goals as “main goals” than men. As expected and in line with previous studies [32], this study showed a trend towards higher expectations regarding working, physical and sports related treatment goals in younger patients (< 65y) and men. In contrast, women chose treatment goals regarding everyday work (e.g. “managing household”, “activities of daily life”) significantly more often. This could be based on the conservative idea of womanhood predominating in the older population that was mostly included in our study. While overall data is scarce, Razmjou et al. [25] showed analogous trends in sex-specific expectations, meaning men having higher levels of expectations of returning to leisure-, recreational-, or work-related activities than women, although using a self-constructed, non-joint-specific questionnaire. Since existing questionnaires do not inquire expectations in these categories in detail, an implementation of the INDICATE Knee Score questionnaire in a different cultural context or an overall younger cohort would be interesting.

BMI had a significant influence on prioritisation of the treatment goal “weight reduction”, suggesting that obese patients expect TKA as a mean for weight reduction, probably through regaining the ability to exercise after surgery. Recent studies described both, weight loss and weight gain after TKA, with substantial effect on post-operative outcome [7, 26, 33]. It is, therefore, of particular importance to guide obese patients to realistic expectations in the pre-operative SDM process.

Younger, obese, and female patients in our cohort tend to rate the reduction of swelling more often as “main goal”, which might be for cosmetic reasons. The significant influence of female sex on the treatment goal “Malalignment” in our cohort might support this assumption. However, to the author’s knowledge no studies exist, describing women having higher cosmetic expectations on surgical outcomes. Surprisingly, “Less dependence on help of others” was more often chosen as main goal from older (> 65-year) patients, while one would usually anticipate that younger patients want to regain autonomy through TKA, as they are expected to care for themselves in a typical community. On the other hand, younger patients might have a larger scale of compensatory mechanisms in dealing with dependence (e.g. better social surroundings, better preparations), and therefore are less cowed of dependence on help of others.

Consistent with a previous study by Lange et al. [18], correlations between treatment goals and related impairments were low on a variety of variables in our cohort. Overall, an increase in correlation from more general to specific variables was observed. Thus, variables like “Knee pain”, “Quality of life”, “Joint mobility”, “Climbing stairs” and “Walking distance” showed low to modest correlation with their respective impairment, suggesting these over-arching treatment goals apply to every patient undergoing TKA and are independent of the impairment prior to surgery. Supporting this presumption is the high correlation in specific variables such as “Sleeping”, “Noise from the joint”, “Swelling” or “Sex life”. Patients with impairments in these categories seem to suffer from a high and specific psychological strain, seeking relief through TKA. These impairments are not typically induced exclusively by knee OA, and hence the probability of improving these impairments through TKA should be discussed during pre-operative SDM.

## Limitations

This study has some limitations. Only patients already enrolled in a TKA surgery process were included, thereby only the expectations of patients with end-stage osteoarthritis are reflected. However, this is typical for patients undergoing TKA and we, therefore, believe this cohort to be representative. The time of elicitation of the questionnaire differed between the participating hospitals, ranging from 1 to 8 weeks before surgery. A potential weakness of the questionnaire could be the mixture of general and specific treatment goals. However, we think this variety reflects the diversity and individuality of patients’ treatment goals for TKA and is helpful for physicians to better understand their patients’ motivation for surgery. In this study, the questionnaire was used paper based; a digital version should be generated for future application. Only patients from Germany were included; therefore, expectations might be different in other countries and cultures.

## Conclusion

Seven treatment goals which were expected by > 90% of patients in our collective can be classified as general treatment goals for TKA. Influence of demographic factors (age, sex, BMI) on pre-operative expectations underline the need for knowing individual patient expectations, allowing physicians to guide their patients to realistic expectations and consequently improve satisfaction after TKA.

## Abbreviations

TKA: Total knee arthroplasty
BMI: Body mass index
OA: Osteoarthritis
SDM: Shared decision-making
HSS: Hospital for special surgery
SD: Standard deviation
